# Development and validation of a surgical-pathologic staging and scoring system for cervical cancer

**DOI:** 10.18632/oncotarget.8245

**Published:** 2016-03-21

**Authors:** Shuang Li, Xiong Li, Yuan Zhang, Hang Zhou, Fangxu Tang, Yao Jia, Ting Hu, Haiying Sun, Ru Yang, Yile Chen, Xiaodong Cheng, Weiguo Lv, Li Wu, Jin Zhou, Shaoshuai Wang, Kecheng Huang, Lin Wang, Yuan Yao, Qifeng Yang, Xingsheng Yang, Qinghua Zhang, Xiaobing Han, Zhongqiu Lin, Hui Xing, Pengpeng Qu, Hongbing Cai, Xiaojie Song, Xiaoyu Tian, Jian Shen, Ling Xi, Kezhen Li, Dongrui Deng, Hui Wang, Changyu Wang, Mingfu Wu, Tao Zhu, Gang Chen, Qinglei Gao, Shixuan Wang, Junbo Hu, Beihua Kong, Xing Xie, Ding Ma

**Affiliations:** ^1^ Department of Obstetrics and Gynecology, Tongji Hospital, Tongji Medical College, Huazhong University of Science and Technology, Wuhan, P.R. China; ^2^ Department of Gynecology and Obstetrics, the Central Hospital of Wuhan, Wuhan, P.R. China; ^3^ Department of Obstetrics and Gynecology, Union Hospital, Tongji Medical College, Huazhong University of Science and Technology, Wuhan, P.R. China; ^4^ Department of Gynecologic Oncology, Hunan Province Tumor Hospital, Changsha, P.R. China; ^5^ Women's Reproductive Health Laboratory of Zhejiang Province, Zhejiang, P.R. China; ^6^ Department of Gynecology and Obstetrics, Qilu Hospital, Shandong University, Shandong, P.R. China; ^7^ Department of Obstetrics and Gynecology, The First Affiliated Hospital, Medical School of Xi'an Jiaotong University, Xi'an, P.R. China; ^8^ Department of Gynecologic Oncology, The Second Affiliated Hospital, Sun Yat-sen University, Guangzhou, Guangdong, P.R. China; ^9^ Department of Obstetrics and Gynecology, Xiangfan Central Hospital, Tongji Medical College, Huazhong University of Science and Technology, Xiangfan, Hubei, P.R. China; ^10^ Tianjin Central Hospital for Gynecology and Obstetrics, Tianjin, P.R. China; ^11^ Department of Gynecologic Oncology, Zhong Nan Hospital, Wuhan University, Wuhan, P.R. China; ^12^ Commercial Vocational Hospital, Wuhan, P.R. China; ^13^ Department of Obstetrics and Gynecology, The First Affiliated Hospital of Henan University of Science and Technology, Luoyang, Henan, P.R. China

**Keywords:** cervical cancer, risk factor, FIGO stage, surgical-pathologic stage, SPSs

## Abstract

**Background:**

Most cervical cancer patients worldwide receive surgical treatments, and yet the current International Federation of Gynecology and Obstetrics (FIGO) staging system do not consider surgical-pathologic data. We propose a more comprehensive and prognostically valuable surgical-pathologic staging and scoring system (SPSs).

**Methods:**

Records from 4,220 eligible cervical cancer cases (Cohort 1) were screened for surgical-pathologic risk factors. We constructed a surgical-pathologic staging and SPSs, which was subsequently validated in a prospective study of 1,104 cervical cancer patients (Cohort 2).

**Results:**

In Cohort 1, seven independent risk factors were associated with patient outcome: lymph node metastasis (LNM), parametrial involvement, histological type, grade, tumor size, stromal invasion, and lymph-vascular space invasion (LVSI). The FIGO staging system was revised and expanded into a surgical-pathologic staging system by including additional criteria of LNM, stromal invasion, and LVSI. LNM was subdivided into three categories based on number and location of metastases. Inclusion of all seven prognostic risk factors improves practical applicability. Patients were stratified into three SPSs risk categories: zero-, low-, and high-score with scores of 0, 1 to 3, and ≥4 (P=1.08E-45; P=6.15E-55). In Cohort 2, 5-year overall survival (OS) and disease-free survival (DFS) outcomes decreased with increased SPSs scores (P=9.04E-15; P=3.23E-16), validating the approach. Surgical-pathologic staging and SPSs show greater homogeneity and discriminatory utility than FIGO staging.

**Conclusions:**

Surgical-pathologic staging and SPSs improve characterization of tumor severity and disease invasion, which may more accurately predict outcome and guide postoperative therapy.

## INTRODUCTION

Cervical cancer is the third leading cause of cancer-related mortality among women worldwide;[1] it results in approximately 275,000 deaths annually.[2] The International Federation of Gynecology and Obstetrics (FIGO) staging system is a widely accepted staging method for cervical cancers.[3, 4] The FIGO system is based solely on clinical examination, and once the clinical stage has been designated it cannot be changed, even if exact surgical or pathologic evidences were confirmed during or after surgery. Therefore, it is inherently inaccurate if patients accompanied by pelvic inflammatory disease, endometriosis, or obesity. Patients within each FIGO stage have markedly different outcomes due to significant discrepancies between clinically determined stage and surgical pathologic findings.[5-7]

According to the FIGO committee, surgical-pathological staging of cervical cancer is problematic in low income countries due to high incidence rates and lower surgical rates,[4] especially in Africa, where the majority of patients are diagnosed in advanced stages and surgical facilities and surgeons are extremely scarce. However, according to a World Health Organization report in 2012, 53.9% of cases worldwide occurred in Asia and 21.2% in Europe and America areas, whereas Africa accounts for only 18.8%.[8] The ten countries with the most cases worldwide were in Northern Asia, Eastern Europe, and America, where incidence rates ranged from 9.4/100,000 to 23.7/100,000, including India, China, Russian Federation, United States, and Japan.[8] Whereas the ten countries with the highest incidence rates (39.9/100,000-56.3/100,000) were mainly in Africa, they account for only a small percentage (3.2%) of cervical cancer cases worldwide (Supplementary Tables 1 and 2).[8] With rapid economic development in Asia, cervical cancer incidence trends and treatment patterns have fundamentally changed.[9] We recently published a 10-year investigation of 10,012 cervical cancer cases in China. [10-13] The vast majority of patients (83.9%) were treated with surgery. Similarly, the majority of cervical cancers are surgically treated in Asia, Europe, and America areas.[14, 15] Therefore, a surgical-pathologic staging system for cervical cancer is appropriate for worldwide implementation and should be proposed as soon as possible.

There is precedent for use of a more practical scoring system in staging of gynecological malignancies.[16-20] For example, patients with malignant trophoblastic tumors simultaneously have several prognostic risk factors and may result in the different outcomes. The introduction of a rational staging system for scoring of malignant trophoblastic tumor has resulted in improved prognostic accuracy to the patients.[21, 22]A surgical-pathologic scoring system (SPSs) can provide important clues to assist clinicians in developing more precise and individualized treatment schemes.

In this study, 4,220 eligible cases were extracted for screening the surgical-pathologic risk factors. The preliminary surgical-pathologic stages and SPSs for cervical cancer were assigned, and a prospective validation was performed to verify the reliability and practical applicability of the new system.

## RESULTS

### Univariate and multivariate analysis of risk factors

The univariate analysis of potential risk factors is shown in Table 1. Eight factors were found to be associated with OS and DFS rates, including lymph node metastasis (LNM) (OS,P=1.49E-32; DFS,P=2.29E-37), parametrial involvement (OS,P=4.23E-18; DFS,P=1.09E-22), histological type (OS,P=3.51E-16; DFS,P=4.13E-12), tumor size > 4cm (OS,P=5.31E-6; DFS,P=1.87E-6), grade2-3(OS,P=0.002; DFS,P=2.62E-4), stromal invasion (OS,P=1.10E-10; DFS,P=2.47E-13), lymph-vascular space invasion (LVSI) (OS,P=1.07E-6; DFS,P=4.64E-10), and vaginal involvement (OS,P=0.003; DFS,P=7.27E-5). Corpus uteri was not associated with the long-term outcomes in this study (OS,P=0.435; DFS,P=0.130). The other three general factors of age, parity, and tumor family history did not exhibit any significant effect on patient prognosis and were therefore discarded from further analysis.

**Table 1:** Univariate and multivariate analyses of risk factors for 5-year OS and DFS rates in cervical cancer patients in Cohort 1

	Total	Univariate analysis						Multivariate analysis					
		5-year OS rates			5-year DFS rates			5-year OS rates			5-year DFS rates		
		Number	Percent	*P* value^a^	Number	Percent	*P* value^a^	Odds ratio	95% CI	P value	Odds ratio	95% CI	P value
Age at diagnosis (yrs)				0.696			0.505	-	-	-	-	-	-
≤ 45	2435	1989	81.7%		1913	78.5%							
> 45	1782	1456	81.7%		1395	78.3%							
Parity				0.356			0.850	-	-	-	-	-	-
≤2	2880	2370	82.3%		2267	78.7%							
>3	1338	1078	80.6%		1044	78.0%							
Tumor family history				0.848			0.304	-	-	-	-	-	-
Negative	3705	3034	81.9%		2918	78.8%							
Positive	515	414	80.4%		393	76.2%							
LNM^b^				1.49E-32			2.29E-37	2.093	1.776-2.467	1.28E-18	2.042	1.754-2.376	2.81E-20
Negative	3491	2954	84.6%		2854	81.8%							
Positive	728	494	67.8%		457	62.7%							
Parametrial invasion				4.23E-18			1.09E-22	1.669	1.387-2.009	5.88E-08	1.737	1.466-2.058	1.83E-10
Not involved	3686	3072	83.3%		2963	80.4%							
Involved	524	366	69.8%		338	64.4%							
Histological types				3.51E-16			4.13E-12	1.666	1.363-2.048	6.55E-07	1.587	1.316-1.973	1.28E-06
SCC	3803	3133	82.4%		3011	79.2%							
AC/ASC	402	310	77.1%		295	73.4%							
Others	14	10	31.8%		4	28.6%							
Grade				0.002			2.62E-4	1.188	1.078-1.310	0.001	1.205	1.102-1.319	4.63E-05
Grade 1	196	177	90.3%		168	85.7%							
Grade 2-3	3549	2860	80.6%		2739	77.2%							
No-grade	475	411	86.5%		404	85.1							
Tumor size				5.31E-6			1.87E-6	1.290	1.097-1.518	0.002	1.270	1.075-1.491	0.002
≤4cm	3315	2754	83.1%		2648	79.9%							
> 4cm	905	694	76.7%		663	73.2%							
Stromal invasion				1.10E-10			2.47E-13	1.251	1.044-1.500	0.015	1.266	1.075-1.491	0.005
≤1/2	3393	2810	82.8%		2715	80.0%							
>1/2	827	638	77.1%		596	72.2%							
Lymph-vascular invasion				1.07E-6			4.64E-10	1.282	0.981-1.675	0.069	1.435	1.135-1.815	0.003
Negative	3917	3228	82.4%		3110	79.4%							
Positive	274	206	75.2%		187	68.0%							
Vaginal involvement				0.003			7.27E-5	-	-	-	-	-	-
Not involved	3207	2635	81.4%		2543	79.3%							
Involved	1007	808	80.2%		763	75.8%							
Corpus uteri				0.435			0.130	-	-	-	-	-	-
Not involved	4136	3383	81.8%		3252	78.6%							
Involved	79	64	81.0%		58	74.3%							

Abbreviation: SCC, Squamous cell carcinoma; AC/ASC, Adeno/adeno-squamous cell carcinoma

a The P value is based on the log-rank test.

b LNM: Lymph node metastasis

Next, the multivariate analysis identified six independent factors associated with both 5-year OS and DFS rates and they were ranked based on the magnitude of their effect as follows: (1) LNM; (2) parametrial involvement; (3) histological type; (4) tumor size > 4cm; (5) grade; and (6) stromal invasion (Table 1). In addition to these factors, LVSI was found to be a risk factor associated only with 5-year DFS rate. In this study, vaginal involvement was not found to affect prognosis.

### Effect of lymph node metastasis on prognosis

Among all of the prognostic parameters in our study, LNM was the strongest determinant of patients’ survival. Both 5-year OS and DFS rates were decreased significantly in patients with LNM in comparison to patients without LNM (Supplementary Figures 1A and 1B; OS, P=1.64E-32; DFS, P=2.82E-37), indicating the positive nodes was defining prognostic factor for the long-term outcomes. Moreover, the 5-year OS and DFS rates decreased concomitant with an increasing number of positive nodes (Supplementary Figures 1C and 1D; OS, P=3.76E-11; DFS, P=1.96E-9; Supplementary Tables 4 and 5). Additionally, when para-aortic lymph nodes metastasis (pLNM) were detected, the 5-year outcomes in these patients were much poor than those of patients without pLNM (Supplementary Figures 1E and 1F; OS, P=4.85E-6; DFS, P=4.66E-5; Supplementary Table 5).

### Establishment of the preliminary surgical-pathologic stages

Based on the analyses above, we created preliminary surgical-pathologic stages for cervical cancer. The goal was to put risk factors into surgical-pathologic stages in order according to their severity and prognostic impact. As shown in Table 2, the basic FIGO framework was retained. In stage I, when the lesions exceeded the criteria defining stage A, but measured tumor diameter was ≤4 cm in size, we divided FIGO IB1 into two surgical-pathologic stages, IB1 (no LVSI and invasion of stroma ≤1/2) and IB2 (positive LVSI and/or invasion of stroma >1/2). The FIGO IB2 criteria were used to define surgical-pathologic stage IB3. In stage II, surgical-pathologic stage IIC was added to include cases in which LNM was identified by pathologists, and the classification was further subdivided, based on the number of positive nodes, into IIC1(cases with 1 or2 LNM) and IIC2 (cases with ≥3 LNM). Similarly, patients with pLNM were classified as surgical-pathologic stage IIC3 (Table 2, Supplementary Tables 4 and 5). Corpus uteri and vaginal involvement were found not to be associated with 5-year OS and DFS rates in this study, and were consequently excluded from the list of criteria used to define surgical-pathologic stage.

**Table 2:** The surgical-pathologic stages of cervical cancer

Stage	Description
I	The carcinoma is strictly confined to the cervix (without pelvic lymph node metastasis; extension to the uterine corpus should be disregarded).
IA	Invasive cancer identified only microscopically (All gross lesions even with superficial invasion are Stage IB cancers.) Invasion is limited to measured stromal invasion with a maximum depth of 5mm^a^ and no wider than 7mm.
IA1	Measured invasion of stroma ≤3 mm in depth and ≤7 mm width.
IA2	Measured invasion of stroma >3 mm and <5 mm in depth and ≤7 mm width.
IB	The lesions greater than stage IA, and the carcinoma not extends beyond the cervix
IB1	No lymph-vascular space and invasion of stroma ≤1/2.
IB2	Lymph-vascular space invasion and/or invasion of stroma >1/2, and pathologically measured tumor diameter ≤ 4 cm in size.
IB3	Pathologically measured tumor diameter > 4 cm in size.
II	The carcinoma extends beyond the uterus, but has not extended onto the pelvic wall or to the lower third of vagina.
IIA	Pathological involvement of up to the upper 2/3 of the vagina. No parametrial involvement and no positive pelvic lymph nodes.
IIA1	Pathologically measured lesions ≤ 4 cm
IIA2	Pathologically measured lesions > 4 cm
IIB	With parametrial pathological involvment but not onto pelvic sidewall and no positive pelvic lymph nodes.
IIC	Pelvic lymph node metastasis
IIC1	1 to 2 positive nodes (without positive para-aortic lymph node)
IIC2	≥3 positive nodes (without positive para-aortic lymph node)
IIC3	Positive para-aortic lymph node
III	The carcinoma has extended onto the pelvic side wall/or involves lower third of the vagina and/or causes hydronephrosis or non-functioning kidney. The tumor involves the lower third of the vagina. All cases of hydronephrosis or non-functioning kidney should be included unless they are known to be due to other causes.
IIIA	Involvement of the lower vagina but no extension onto pelvic side wall.
IIIB	Extension onto the pelvic side wall, or hydronephrosis/non-functioning kidney.
IV	The carcinoma has extended beyond the true pelvis or has clinically involved the mucosa of the bladder and/or rectum.
IVA	Spread to adjacent pelvic organs.
IVB	Spread to distant organs.

a The depth of invasion should not be more than 5 mm taken from the base of the epithelium, either surface of glandular, from which it originates.

### Construction and validation of spss

In surgical-pathologic staging, two important parameters, histological type and grade, were not covered. To provide a more accurate and convenient system for evaluation of patients’ prognosis, the SPSs was proposed. Regression coefficients (β) yielded a statistical weight for the contribution of each factor (Table 3). The total score was generated by superposition when a single patient had more than one risk factor. We set up a rank of optimal cutoff points (Table 4): SPSs A (2 and 4, score 0-1; 2-3; ≥4), SPSs B (3 and 4, score 0-2; 3; ≥4), SPSs C (1 and 4, score 0; 1-3; ≥4), and SPSs D (1 and 5, score 0; 1-4; ≥5). The SPSs C system exhibited the best monotonicity of gradient, based on LR*χ^2^* (highest homogeneity, 192.0) and liner trend*χ^2^* (highest discriminatory score at 8 years, 28.2). The Akaike information criterion was the lowest if choosing SPSs C (Table 4).

**Table 3:** The surgical-pathologic scores in patients with cervical cancer in Cohort 1

Risk factors	5-year OS rates		5-year DFS rates	
	β	Score	β	Score
Lymph node metastasis	0.739	4	0.714	4
Parametrial invasion	0.512	2	0.552	2
Histological types: Others^a^	0.511	2	0.462	2
AC/ASC^b^		1		1
Tumor size (> 4cm)	0.255	1	0.239	1
Grade (Grade 2-3)	0.248	1	0.361	1
Stromal invasion (>1/2)	0.224	1	0.236	1
Lymph-vascular space invasion	0.172	1	0.187	1

a Others: small cell carcinoma, neuroendocrine carcinoma, carcinoid tumor

b AC/ASC: Adeno/ adeno-squamous cell carcinoma

**Table 4:** Comparison of prognostic stratification of different cervical cancer staging and scoring systems

Systems	Groups	LR χ^2^ test^a^	AIC^b^	Linear tread χ^2^ at 5 years	Linear tread χ^2^ at 8 years
SPSs A^c^	Scores: 0-1; 2-3; ≥4	155.2	12142.1	138.7	24.7
SPSs B^d^	Scores: 0-2; 3; ≥4	187.7	12123.9	155.0	20.4
SPSs C^e^	Scores: 0; 1-3; ≥4	192	12101.4	154.4	28.2
SPSs D^f^	Scores: 0; 1-4; ≥5	188.2	12108.2	139.2	23.5
Surgical-pathologic stage	-	152.4	12153.3	125.8	15.7
FIGO stage	-	54.2	12238.8	55.9	7.3

a LR, likelihood ratio

b AIC, Akaike information Criteria

c SPSs A, Scoring system with cutoff points of 2 and 4

d SPSs B, Scoring system with cutoff points of 3 and 4

e SPSs C, Scoring system with cutoff points of 1 and 4

f SPSs D, Scoring system with cutoff points of 1 and 5

FIGO stage, International Federation of Gynecology and Obstetrics staging system stratified with IA, IB, IIA, IIB, and III; SPSs, Surgical-pathologic stage stratified with IA, IB, IIA, IIB, IIC, and III

**Figure 1:**
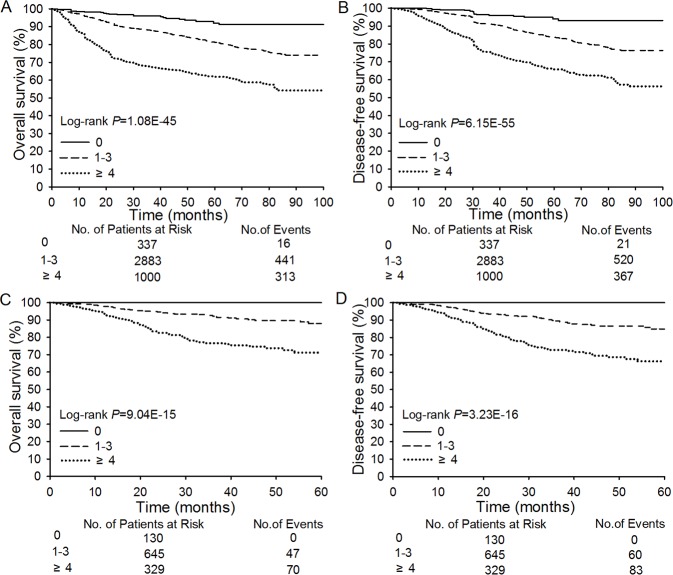
Overall Survival and Disease-free Survival According to Risk Scores Panel **A.-B.** shows overall survival (OS) rates and disease-free survival (DFS) rates for patients in the zero score (0, *n* = 337), low-score (1-3; *n* = 2,883), and high-score (≥ 4; *n* = 1,000) groups in Cohort 1. Panel **C.-D.** shows OS and DFS rates for patients in the zero score (0,*n* = 130), low-score (1-3; *n* = 645), and high-score (≥ 4; *n* = 329) groups in Cohort 2.

Based on serial inference and optimization, the patients could be stratified by SPSs C array into three risk categories: zero risk, low risk, and high risk. To further validate the reliability of SPSs, all patients in Cohort 1 were classified into three score groups based on the sum of their risk factors. The 5-year OS and DFS outcomes in these three groups showed significant decreases associated with increased scores, and differences among three groups were found to be significant (Figure 1A and 1B, P=1.08E-45 and P=6.15E-55; Supplementary Table 6). Next, we performed external validation. To reach this purpose, a total of 1,104 cases in Cohort 2 were enrolled, from 2009 to 2013, in a prospective study. The results from this Cohort 2 study completely confirmed the results of the Cohort 1 study. (Figure 1C and 1D; P=9.04E-15 and P=3.23E-16; Supplementary Table 6).

### Comparison of figo staging, surgical-pathologic staging, and spss

In this study, we compared FIGO staging, surgical-pathologic staging, and SPSs (SPSs C: zero, low, and high scores) by their monotonicity and discriminatory ability. The best monotonicity and discrimination was provided by SPSs, followed by surgical-pathologic staging and then FIGO staging (Table 4, LR*χ^2^*: 192.0, 152.4, and 54.2 respectively; linear trend*χ^2^* at 8 year: 28.2, 15.7, and 7.3, respectively), SPSs was also showed the lowest Akaike information criterion among three systems (Table 4, AIC: 12101.4, 12153.3 and 12238.8, respectively), indicating that SPSs was the most informative system with respect to explaining or predicting the prognosis of patients with cervical cancer.

## DISCUSSION

The Cohort 1 study included multicenter samples. Whereas the majority of parameters we concerned have also been considered in previous studies, [23-25] However, our study is the first to provide definitive, statistically significant findings based on a large cohort. External validation was performed with data from Cohort 2, a high-quality single center data set. The use of patient data from China in the Asia-Pacific region is particularly relevant and informative considering both the large population size and the high prevalence of cervical cancer, which makes this region the site of the most cases of cervical cancer worldwide (Supplementary Table 2).

Among the seven cervical cancer risk factors examined, LNM was found to be the strongest prognostic factor; this association has been extensively documented, [26-28] but lymph node status is still not considered within the FIGO staging system. Our present statistical analysis provides new insight into the relevance of LNM to long-term outcomes. Here we provide compelling evidence that LNM should be introduced into the surgical-pathologic staging system, and propose sub-classification of surgical-pathologic stages IIC1, IIC2, and IIC3 based on the number of positive nodes and presence or absence of pLNM (Table 2). A survey of pLNM is listed in Supplementary Table 7. Parametrial involvement, tumor size, and stromal invasion were found to be additional key risk factors for prognosis. All of the parameters were re-staged based on their impact values. LVSI was found to be a marginal independent risk factor and was therefore also included. In contrast, corpus uteri and vaginal involvement were not found to be associated with long-term outcomes. We presume that this is due to the ability to completely surgically excise both types of lesion, such that they probably lose any long-term effects on prognosis after surgical removal. However, there is no consensus on this view [28-31]. Therefore, we have not included these two parameters in the new surgical-pathologic staging system to date.

We noticed that two crucial surgical-pathologic parameters, histological types and grades, were not utilized in defining the surgical-pathologic stages. To provide a more reliable and convenient system to predict patient outcome, we propose a new scoring system for cervical cancer. There are several successful examples of scoring systems in gynecologic practice, such as those used for gestational trophoblastic neoplasms and endometriosis. [17, 18, 32, 33] In our system, the score value was compiled by statistical weight of each individual factor, and then optimized SPSs risk categories were proposed in order to introduce a logical standard for the classification of cervical cancer and for use in setting guidelines for post-operative treatment. External-validation as well as homogeneity and discriminatory analysis indicate that SPSs has the most value of all of the three systems with respect to prognostic interpretation in cervical cancer.

In conclusion, the prognostic parameters used in our study are derived from both surgically-derived data and pathologic examination, which most objectively reflect the patient's real condition. The surgical-pathologic staging and SPSs presented here may enable clinicians to more precisely predict the prognosis of patients and to standardize post-surgical therapeutic recommendations for defined sets of cervical cancer patients (e.g. non treatment for zero-score group; adjuvant external radiotherapy for low-score group; and concurrent chemoradiotherapy for high-score group), ultimately improving patient survival.

## PATIENTS AND METHODS

### Patients

The study was based on two cohorts. Cohort 1 comprised 4,220 eligible patients of 10,087 inpatient cases during 2002 to 2008, extracted from the Cervical Cancer Database v1.10 (http://clinicaltrials.gov, Supplementary Figure 2), which has been described previously. [11, 12] Cohort 2 comprised 1,104 inpatient cases enrolled in a prospective study at Tongji Hospital from 2009 to 2013. All cases in both cohorts were FIGO stage IA-III cervical cancer patients. All were treated surgically. Baseline of patient characteristics is summarized in Supplementary Table 3. Cohort 1 was used as the training set to screen surgical-pathologic risk factors for establishment of surgical-pathologic stages and development of the SPSs, and Cohort 2 was used for external validation of the SPSs. Pathological diagnoses were confirmed by two pathologists in both cohorts. Patients who met any of the following criteria were excluded: over 70 years of age, other serious complicating disease, or prior malignant disease. There was no overlapping of patients between the two cohorts. The protocol was approved by the Ethics Committee of Tongji Hospital, Tongji Medical College, Huazhong University of Science and Technology, P. R. China. All patients in Cohort 2 provided written informed consent.

### Clinical assessment

Previous reports describe the details of the Cohort 1data sources and the methods used for this analysis.[11, 12] Briefly, data were collected from patient records by trained gynecological oncology staff using standardized data collection and quality control procedures. The collected data included demographics; clinical and pathologic tumor characteristics; treatment regimens; physical, gynecologic, cytological, and pathological observations from patient examinations; and outcomes.

All participants in Cohort 2 were evaluated by trained gynecological oncology staff. A structured questionnaire was collected at recruitment, covering socio-demographic characteristics, cigarette smoking, alcohol consumption, family history of cancer, and history of menopause, pregnancy, delivery, and use of oral contraceptives. At the time of enrollment and during follow-up examinations the following tests were performed to determine patient status before and after treatment: gynecological examination, liquid-based thin-layer cytology and human papilloma virus tests, ultrasonographic evidence or computer tomography/magnetic resonance imaging, and serological tests including squamous cell carcinoma antigen.

### Treatment and follow-up

Generally, patients at early FIGO stages without positive pathological findings received no further treatment except for regular follow-up examinations. Patients at later FIGO stages or with pathological findings received adjuvant radiotherapy or concurrent chemoradiotherapy (CCRT) according to Radiation Therapy Oncology Group (RTOG) guidelines. Follow-up examinations were advised for all patients every three months for the first two years, every six months for the next four years, and yearly thereafter. A few patients in the study did not complete follow-up, and were excluded from survival analysis.

### Statistical analysis

Statistical analyses were performed using the SPSS 13.0 software package. Categorical variables are presented as frequencies and percentages, and continuous variables are presented as means ± standard deviation (SD). P value of less than 0.05was considered to indicate statistical significance. Overall survival (OS) and disease-free survival (DFS) rates were calculated using the Kaplan-Meier method, and the log-rank test was used to compare survival curves.

The developmental model comprised three steps. Firstly, univariate and multivariate Cox proportional-hazards models were used to determine the contribution of the variables (P<0.05). The Cox proportional hazard model with stepwise approach was used to identify independent predictors of OS and DFS rates in multivariate analyses. Second, a simple risk score was devised using significant variables obtained from the stepwise multivariate analysis (P<0.05).[34] The score was defined as the weighted sum of those variables (rounded to the nearest integer). The regression coefficients retained in the clinical score model provided a statistical weight for the contribution of each factor to the overall risk of long-term outcomes. The scoring system was then adjusted by the addition of a constant across all scores to ensure that none of the values were below zero.[23, 35] Third, cutoff values were applied to categorize the scores into three groups: zero, low-, and high-score groups. We used the ordinary prognostic score in the likelihood ratio (LR) test, rather than using dummy variables.[36, 37] The Cox regression results were expressed using the Akaike information criterion.[38] The linear trend*χ^2^* test was used to evaluate the discriminatory ability and the monotonicity of survival gradients.[36, 37] The 5- and 8-year rates were determined using the training cohort data, and the cutoff points providing the best homogeneity, discriminatory ability, and monotonicity of gradients were chosen for use in the final scoring system. Furthermore, a comparison between FIGO staging, surgical-pathologic staging, and the SPSs was performed. Finally, the risk-scoring model was validated by comparing the OS and DFS rates among the three risk-based subgroups in Cohort 2.

### Highlights

- The current International Federation of Gynecology and Obstetrics (FIGO) staging system do not consider surgical-pathologic data.
- We propose a more comprehensive and prognostically valuable surgical-pathologic staging and scoring system (SPSs).
- Surgical-pathologic staging and SPSs show greater homogeneity and discriminatory utility than FIGO staging.

## SUPPLEMENTARY MATERIAL FIGURES AND TABLES


