# Supplementary material for: Development and validation of a surgical-pathologic staging and scoring system for cervical cancer
Source: Oncotarget. 2016 Mar 21;7(15):21054–63. doi: 10.18632/oncotarget.8245 (PMC4991512; doi:10.18632/oncotarget.8245)
Supplement: Supplementary file 1 [file oncotarget-07-21054-s001.pdf]

Development and validation of a surgical-pathologic staging and scoring system for cervical cancer

Supplementary Material

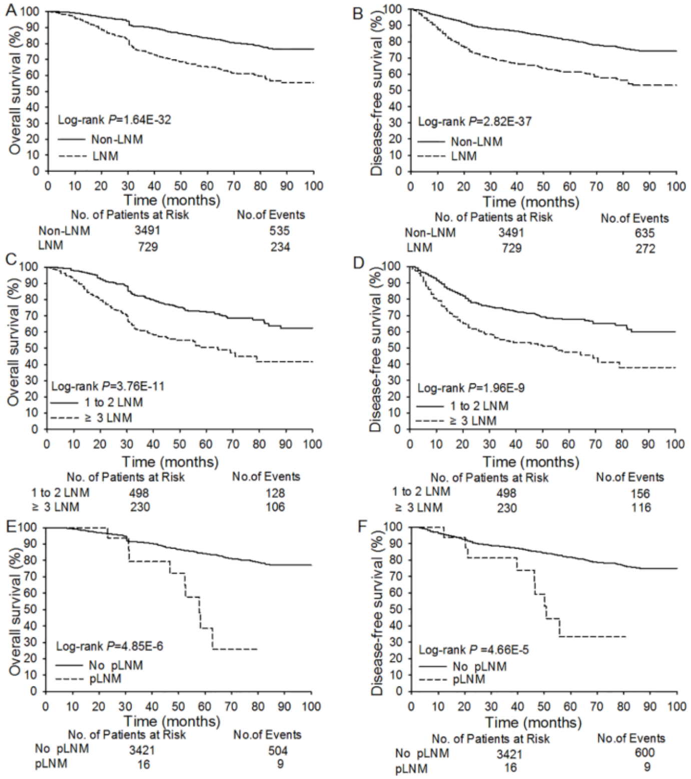

Supplementary Figure 1. Overall Survival and Disease-free Survival According to Lymph Node Metastasis in Cohort 1.

Panel A-B shows overall survival (OS) and disease-free survival (DFS) of patients in the Non-Lymph Node Metastasis (non-LNM) group (n = 3,491) and the LNM group (n = 729). Panel C-D shows OS and DFS of patients in the group with 1 to 2 LNM (n = 498) and the group with ≥ 3 LNM (n = 230). Panel E-F shows DFS and OS for patients in the Non- Para-aortic Lymph Nodes Metastasis (pLNM) group (n = 3,421) and the pLNM group (n = 16).

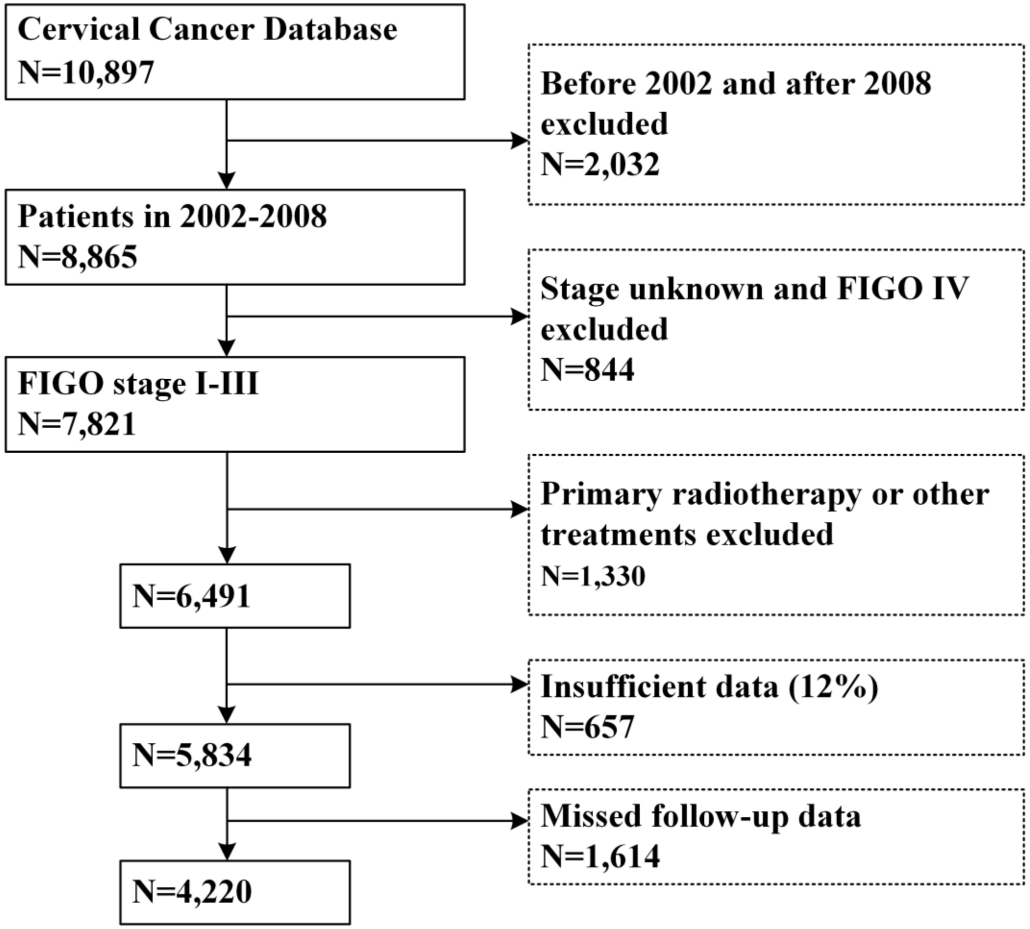

Supplementary Figure 2. Cohort 1 Patient Selection in the Present Study.

Supplementary Table 1 Ten countries with the top highest incidence of cervical cancer according to the statistics data of GLOBOCAN 2012[a]

**Countries**	**Cases** (10^3)	**Incidence** ($/10^5$)	**Deaths** (10^3)	**Mortality** ($/10^5$)
Malawi	3.7	46.5	2.3	29.2
Mozambique	5.6	44.8	4.1	32.4
Guyana	0.2	42.7	0.1	18.8
Suriname	0.1	40.2	0.04	16.5
Bolivia	2.0	39.5	0.8	16.5
Romania	4.3	39.4	1.9	17.3
Comoros	0.2	39.4	0.1	24.0
Fiji	0.2	37.5	0.1	19.6
Swaziland	0.2	36.0	0.1	19.1
Lithuania	0.6	34.9	0.2	12.5

[a] GLOBACAN 2012 database, Cervical Cancer Incidence and Mortality Worldwide in 2012 Summary[3]

Supplementary Table 2 Ten countries with the top most cases of cervical cancer according to the statistics data of GLOBOCAN 2012[a]

**Countries**	**Cases** (10^3)	**Incidence** ($/10^5$)	**Deaths** (10^3)	**Mortality** ($/10^5$)
India	122.8	20.2	67.5	11.1
China	61.7	9.4	29.5	4.5
Indonesia	20.9	17.0	9.5	7.7
Brazil	18.5	18.4	8.4	8.3
Russian Federation	15.3	20.0	7.4	9.6
Nigeria	14.1	17.1	8.2	10.0
Mexico	14.0	23.7	4.8	8.1
The United States	13.0	8.1	6.6	4.1
Bangladesh	12.0	15.9	6.6	8.7
Japan	9.4	14.5	3.6	5.6

[a] GLOBACAN 2012 database, Cervical Cancer Incidence and Mortality Worldwide in 2012 Summary[3]

Supplementary Table 3 The baseline of patients in this study

	Characteristics	Cohort 1 (n=4,220)<br>No of patients (%)	Cohort 2 (n=1,104)<br>No of patients (%)
Age at diagnosis (yrs)	Mean	43.86	45.29
	SD	8.81	7.79
	≤ 45	2437(57.7%)	590(54.3%)
	> 45	1780(42.2%)	514(46.6%)
	Unknown	3(0.1%)	0(0.0%)
Parity	≤2	3674(87.1%)	777(70.4%)
	>3	545(12.9%)	287(26.0%)
	Unknown	1(0.0%)	0(0.0%)
Tumor family history	Negative	3706(87.8%)	973(88.1%)
	Positive	618(14.6%)	131(11.9%)
	Unknown	0(0%)	0(0%)
FIGO stage	IA	289(5.6%)	85(7.7%)
	IB1	2165(41.6%)	390(35.3%)
	IB2	1191(22.9%)	128(11.6%)
	IIA	969(18.6%)	169(15.3%)
	IIB	560(10.8%)	303(27.4%)
	III	22(0.5%)	29(2.6%)
	Unknown	1(0 %)	0(0%)
Histological types	SCC[a]	4686(90.1%)	942(85.3%)
	AC/ ASC	491(9.4%)	141(12.8%)
	Other carcinoma	22(0.4%)	17(1.5%)
	Unknown	1(0%)	4(0.4%)

Histological grade	Grade 1	249(4.9%)	82(7.4%)
	Grade 2-3	4343(83.5%)	939(85.1%)
	No-Grade	608(11.7%)	83(7.5%)
	Unknown	0(0%)	0(0%)
Tumor size	≤4cm	4172(80.2%)	772(69.9%)
	> 4cm	1028(19.8%)	332(30.1%)
	Unknown	0(0%)	0(0%)
LNM[b]	Negative	4344(83.6%)	942(85.3%)
	Positive	855 (16.4%)	159(14.4)
	Unknown	1(0.0%)	3(0.3%)

[a]SCC: Squamous cell carcinoma; AC/ASC: Adeno/ adeno-squamous cell carcinoma; [b]LNM: Lymph node metastasis

Supplementary Table 4. 5-year OS and DFS rates stratified by lymph nodes status in FIGO stages in Cohort 1.

FIGO stages	Lymph nodes status	Death/Total (No. of Patients)	5-year OS rates (%)	SE (%)	P value[a]	Recurrence/Total (No. of Patients)	5-year DFS rates (%)	SE (%)	P value[a]
I	No positive nodes	379/2532	84.2	0.8	<0.001	434/2532	82.2	0.9	<0.001
	1 to 2 positive nodes	74/301	74.0	2.8		92/302	69.0	2.9	
	3 or more positive nodes	48/111	52.4	5.5		52/111	50.6	5.3	
	Para-aortic LNM[b]	4/9	42.9	18.7		4/9	44.4	18.9	
II	No positive nodes	151/943	81.9	1.5	<0.001	194/943	78.2	1.5	<0.001
	1 to 2 positive nodes	52/197	69.3	3.9		61/197	66.8	3.8	
	3 or more positive nodes	55/109	48.3	5.1		61/109	43.1	5.1	
	Para-aortic LNM[b]	5/7	28.6	17.1		5/7	28.6	17.1	
III		13/22	39.1	11.8		15/22	27.3	11.1	

[a]The P value is based on Kaplan-Meier method; [b]LNM: Lymph node metastasis

Supplementary Table 5. 5-year OS and DFS rates stratified by lymph nodes status in FIGO stages in Cohort 1.

Lymph nodes status	FIGO stages	Death/Total (No. of Patients)	5-year OS rates (%)	SE (%)	P value[a]	Recurrence/Total (No. of Patients)	5-year DFS rates (%)	SE (%)	P value[a]
No positive nodes	IA	6/179	96.5	8.1	<0.001	7/179	96.2	1.9	<0.001
	IB	372/2353	83.4	0.9		427/2353	81.4	0.9	
	IIA	90/625	83.8	1.8		118/625	80.1	1.8	
	IIB	61/318	78.2	2.6		76/318	74.5	2.7	
1 to 2 positive nodes	IA	0/11	-	-	<0.001	0/11	-	-	<0.001
	IB	74/290	73.2	2.9		92/291	67.9	2.9	
	IIA	25/105	69.5	5.6		29/105	68.2	5.2	
	IIB	27/92	69.2	5.3		32/92	65.6	5.4	
≥3 positive nodes	IA	-	-	-	<0.001	-	-	-	<0.001
	IB	48/111	52.4	5.5		52/111	50.6	5.3	
	IIA	24/57	56.5	7.0		27/57	53.5	6.7	
	IIB	31/52	40.9	7.0		34/52	34.8	6.9	
Para-aortic LNM	1B1-III	9/16	38.5	14.2		9/16	33.2	13.8	

[a]The P value is based on the Kaplan-Meier method.

Supplementary Table 6. 5-year OS and DFS rates stratified by SPSs in both Cohorts.

FIGO stages	score categories	Death/Total (No. of Patients)	5-year OS rates (%)	SE (%)	P value[a]	Recurrence/Total (No. of Patients)	5-year DFS rates (%)	SE (%)	P value[a]
Cohort 1	zero score	17/338	94.1	1.6	P=1.08E-45	22/338	92.0	1.8	P=6.15E-55
	low-score (1-3)	442/2883	83.7	0.8		521/2883	81.2	0.8	
	High-score (≥4)	313/999	65.8	1.7		367/999	61.8	1.7	
Cohort 2	zero score	0/130	100.0	0.0	P=9.04E-15	0/130	100.0	0.0	P=3.23E-16
	low-score (1-3)	47/645	88.0	2.0		60/645	84.9	2.1	
	High-score (≥4)	73/329	71.2	3.1		86/329	66.3	3.4	

[a]The P value is based on the Kaplan-Meier method.

Supplementary Table 7 Reviews about 5-year OS rates of patients with Para-aortic LNM[a].

Follow-up time	Author	Cases	FIGO stage	OS rates	Journals
5-year	This study 2014	16	1B1-III	38.5%	
	Vigliotti, et al. 1992	43	IB-IV	32%	Int J RadiatOncolBiolPhys
	Husseinzadeh, et al. 1994	17	IB-IIIB	12%	Gynecologic Oncology
	Fine, et al. 1995	53	II-III	16-19%	Int J RadiatOncolBiolPhys
	Kim, et al. 1998	43	IB-IVB	24%	GynecolOncol
	Grigsby, et al. 2001	43	I-III	32%	Int J RadiatOncolBiolPhys
	Houvenaeghel, et al. 2006	5	IB–IVA	40%	GynecolOncol
	Rouzier, et al. 2005	30	IB-II	24%	Eur J SurgOncol
	Hacker, et al. 2013	5	IB2	20%	ObstetGynecol
	Manetta, et al. 1986	<115	IB-IV	20%	GynecolOncol
	Grigsby, et al. 2001	30	I-IV	29%	Int J RadiatOncolBiolPhys
4-year	Berman, et al. 1984	98	IB-III	28%	GynecolOncol
	Varia, et al. 1998	86	II-IVA	39%	Int J RadiatOncolBiolPhys
3-year	Morice, et al. 1999	32	IB-IIB	35%	Gynecologic Oncology
	Morice, et al. 2000	14	IB-IIA	40%	Int J Gynecol Cancer
	Delpech, et al. 2007	13	IB2-II	28%	Annals of Surgical Oncology
	Touboul, et al. 2010	19	IB2-IVA	29%	The Oncologist
	Gouy, et al. 2013	29	IB2-IVA	41%	Journal Of Clinical Oncology

[a]LNM: Lymph node metastasis
